# Navigating health policies and programs in India: exploring opportunities to improve rare disease management and orphan drug research

**DOI:** 10.1186/s13023-024-03377-6

**Published:** 2024-11-29

**Authors:** Sangita Mishra, Deepa Bhat, M. P. Venkatesh

**Affiliations:** 1 Centre of Excellence in Regulatory Sciences (CeRES), Department of Pharmaceutics, JSS College of Pharmacy, JSS Academy of Higher Education and Research, SS Nagara, Mysuru, Karnataka 570015 India; 2 Department of Anatomy, JSS Medical College, JSS Academy of Higher Education and Research, SS Nagara, Mysuru, Karnataka 570015 India; 3 Faculty of Pharmaceutical Sciences, UCSI University, Kuala Lumpur, Malaysia

**Keywords:** Indian rare disease management, Health policies in India, Indian rare disease policy, Indian healthcare programs, Orphan drugs

## Abstract

**Background:**

Rare disease (RD) management and orphan drug development in India face various hurdles regarding the implementation and adoption of comprehensive policies, lack of dedicated regulatory frameworks, and absence of epidemiological data. Current rare disease policy focuses more on strengthening the diagnostics and lacks a proper comprehensive treatment framework to ensure favorable clinical outcomes. Indian patients are largely excluded from global orphan drug clinical trials. This further alienates patients from access to rare disease treatment and available treatments come at high cost. This review-based study assesses the landscape of health policies and programs in India through a review of literature and guidelines, to identify strategic opportunities and recommendations for enhancing the overall care and support for the Rare Disease (RD) patient population and improving the orphan drug research ecosystem in India.

**Discussion:**

The absence of specific regulations, shortage of healthcare resources, budget constraints, competing health priorities, lack of patient data, and insufficient research incentives discourage orphan drug development and global clinical trial inclusion, resulting in treatment inaccessibility and high costs. The Indian Government introduced the National Policy for Treatment of Rare Diseases (NPRD) to address these challenges. Several initiatives have been introduced to attract stakeholders with government-funded research, grants, incentives, and accelerated regulatory approvals of novel therapies that can ensure timely prevention and treatment of rare diseases. The National RD Registry by the Indian Council of Medical Research (ICMR) aims to provide prevalence data. Innovative approaches are required to improve rare disease management and promote orphan drug research. This will ensure the accessibility and affordability of life-saving therapeutics for India’s rare disease patients.

**Conclusion:**

An integrated RD management and orphan drug research framework focusing on robust data management, patient-oriented policies to improve the treatment landscape, flexible regulations, strengthening rare disease registry with clinical and diagnostic data, and a favorable research ecosystem to promote indigenous research catering to the Indian population, will improve the treatment landscape and orphan drug research and development in India. This will ensure timely availability of therapeutics at affordable prices.

## Background

Rare Diseases (RDs) impact fewer people than highly prevalent diseases, making it difficult to estimate the target patient population. Despite their low prevalence and individual rarity, RD afflicts a sizable population in every nation, which ranges between 6% and 8% of a country’s population as per existing research and estimates [1, 2]. 6000–8000 rare diseases are estimated to occur globally, and new rare diseases are frequently described in the medical literature, which adds to the list of already established and studied diseases. Since 80% of RDs are of genetic origin, children are disproportionately affected [3]. 

India has approximately 450 known RDs, including autoimmune disorders, lysosomal storage diseases, sickle cell disease, and muscular dystrophies [4]. However, collecting accurate epidemiological data faces numerous hurdles due to large demographic diversity, variance in disease phenotypes, inadequate awareness, underdeveloped research infrastructure, and limited access to state-of-the-art medical and data management technologies and methods [5]. The availability of timely diagnosis and therapies for various conditions related to RDs is hindered by limited facilities, lack of diagnostic expertise, and delayed assessment. The high cost of treatment and absence of robust payer guidelines significantly impact the affordability of groundbreaking therapies for RD patients. This required Governmental intervention, pricing subsidy, and reimbursement of treatment costs imposing a considerable macroeconomic burden on healthcare expenditure [6]. RDs were not considered a significant health concern until 2017 when the Government of India (GoI) acknowledged the severity of RDs and the Ministry of Health and Family Welfare (MoHFW), released NPRD [7] which was revised in 2021. It acknowledged that the issues plaguing the current rare disease management and orphan drug availability in India need to be holistically addressed, with a focus on quality and affordable treatment for RDs [8]. The policy suggested the need for strategic planning for RD management, proposed various initiatives, and encouraged coordinated efforts among multiple stakeholders to achieve equitable, affordable, and quality RD healthcare and to improve the RD management landscape [9]. 

There are ongoing initiatives in the scientific and non-scientific communities on RD management in India. There have been suggestions for integrating various healthcare programs in India to form a centralized rare disease management ecosystem as expected in NPRD. This study focuses on existing policies and guidelines on various healthcare programs, Government healthcare initiatives, and rare disease management systems in India. It aims to identify and aggregate evidence on the rare disease management landscape and the challenges in providing adequate care and coverage to the Indian patient population. It also analyses initiatives undertaken at both the Central and state government levels to address the needs of RD patients and initiatives undertaken to promote research and development of orphan drugs. The outcomes of the reviews were synthesized and assessed to recommend policy considerations and future directions through an integrated framework of rare disease management and orphan drug research and development infrastructure in India.

## Methodology

The selection of relevant literature used in this study was based on the Preferred Reporting Items for Systematic Reviews and Meta-Analyses (PRISMA) guidelines. Refer PRISMA Checklist as presented in the Supplement document for details. The criteria for selecting studies and documents for the review work are shown below:

### Eligibility criteria

We included systematic or narrative reviews, meta-analyses, interviews, policy documents, and guidelines based on which our study was undertaken. We focused on the Indian population and interventions related to rare diseases and the orphan drug research landscape. For the analysis, we employed specific inclusion and exclusion criteria which are discussed in Table 1.

**Table 1:** Eligibility criteria of study selection

Inclusion Criteria	Exclusion Criteria
**Language**: Studies and publications in the English language were included.	**Non-English Documents**: Any document that was not in the English language was excluded.
**Credible Sources**: Documents from credible sources, such as the MoHFW (Ministry of Health and Family Welfare) and the GoI (Government of India) or related agencies were included.	**Contents not specific to Rare Diseases**: Documents not specifically addressing the challenges around rare disease management and healthcare programs were excluded.
**Policy-Related Review Articles and Interviews**: Reviews and interview-based qualitative studies related to health policies in India, rare disease management in India, and general information on global rare disease scenarios found in academic journals were included.	**Studies focused on specific rare diseases**: Studies solely focused on specific rare diseases (e.g., case reports, treatment studies) were excluded.
**Publication Timeframe**: Materials published from January 2000 to the current date were included.	**Insufficient coverage**: Studies that did not explore the specific aspects of rare disease management and orphan drug research in India were excluded.
**Focus on Rare Diseases in India**: Studies specifically focusing on rare diseases and orphan drug research in India were included.	Studies that were not peer-reviewed were excluded.
**Health Policies and Programs in India**: Studies exploring health policies and programs relevant to rare disease management in the Indian scenario were included.	
**Peer-Reviewed Journals**: Studies published in the English language in peer-reviewed journals were included.	

### Identification of studies

The following search query was used to identify relevant studies, policy documents, and materials from online bibliographic repositories like PubMed, SCOPUS, BMC, Web of Science, and Internet search. Keyword searches were performed within titles, abstracts, and author keywords for comprehensive coverage of research literature.

Keywords covered the below terms for searches on rare disease management in India:

- Rare Diseases in India, Rare Disease management in India.
- Health policies in India, Government programs for Rare Diseases, Ministry of Health and Family Welfare, Government of India, and Policies on Rare Diseases in India.
- Systematic review, narrative review, meta-analysis, interviews, guidelines, health missions and programs.

The following search query was utilized for performing the searches:

((“Rare Diseases in India” OR “Rare Disease Management in India”) AND ((“Health policies in India” OR “Government programs for Rare Diseases” OR " Ministry of Health and Family Welfare Guidelines” OR “Government of India Health policies” OR “Policy-related articles on Rare Diseases in India”)) AND (“Systematic review” OR “narrative review” OR “meta-analysis” OR “interviews”))

Keywords covered the below terms for searches on orphan drug research in India:

- Orphan Drugs in India, Orphan Drug Research in India, Orphan Drug Clinical Development in India.
- CDSCO guidelines, Drug Approval, Government of India, ICMR initiatives.
- Systematic review, narrative review, meta-analysis, interviews, guidelines.

The following search query was utilized for performing the searches:

((“Orphan Drugs in India” OR “Orphan Drug Research in India” OR “Orphan Drug Clinical Development in India”) AND ((“CDSCO guidelines” OR “Drug Approval” OR “Government of India” OR “ICMR initiatives”)) AND (“Systematic review” OR “narrative review” OR “meta-analysis” OR “interviews” OR “guidelines”))

### Inclusion and exclusion criteria

#### Study selection

We tailored data extraction to fit the study’s qualitative and narrative nature, systematically retrieving meta-data like document details, publication dates, objectives, designs, policies, shortcomings, opportunities, and findings. Full-text articles of potentially relevant studies were retrieved and further assessed for eligibility. Two independent reviewers (SM and MP) ensured the quality and accuracy of retrieved information by reviewing the principal aspects covered in the documents. A third reviewer (DB) confirmed the adequacy of the contents and quality of the documents used in the review. The selection outcomes focused only on studies and texts that explicitly discussed the key findings of rare disease management in India, the current landscape, policies, and initiatives as well as those studies that identified areas of improvement and suggested scientific and policy initiatives that can help improve the rare disease management system and orphan drug clinical research ecosystem in India. This helped to narrow down only relevant materials that could be used for the study. 190 relevant literature and guidelines were identified and retrieved through the initial search, which was further assessed for content and quality as per inclusion criteria and 25 full-text articles and 63 guidelines, policy-related documents, online articles, and government-issued updates were selected to conduct the final study.

### Information extraction and assessment

Information extraction and assessment involved systematically summarizing and interpreting the findings from the included studies. To facilitate analysis, the studies were categorized based on the specific health policies and programs they address. This categorization allowed the authors to identify key themes and patterns across the literature. The results section summarizes the key findings from the reviewed literature and policy and guideline documents, which were used to address the study objectives.

### Synthesis

A narrative synthesis approach was employed to analyze the extracted data. This was carried out by the authors collaboratively. The narrative synthesis took into account the specificities of each of the studies and the target outcomes of the health policies and programs to come up with a holistic framework for developing an integrated rare disease management and orphan drug ecosystem in India. This analysis and the related outcome aimed to identify opportunities for improvement in rare disease management and orphan drug clinical research within the Indian healthcare setup and research ecosystem. These opportunities were discussed and presented in the context of the identified policies and programs.

**Fig. 1:**
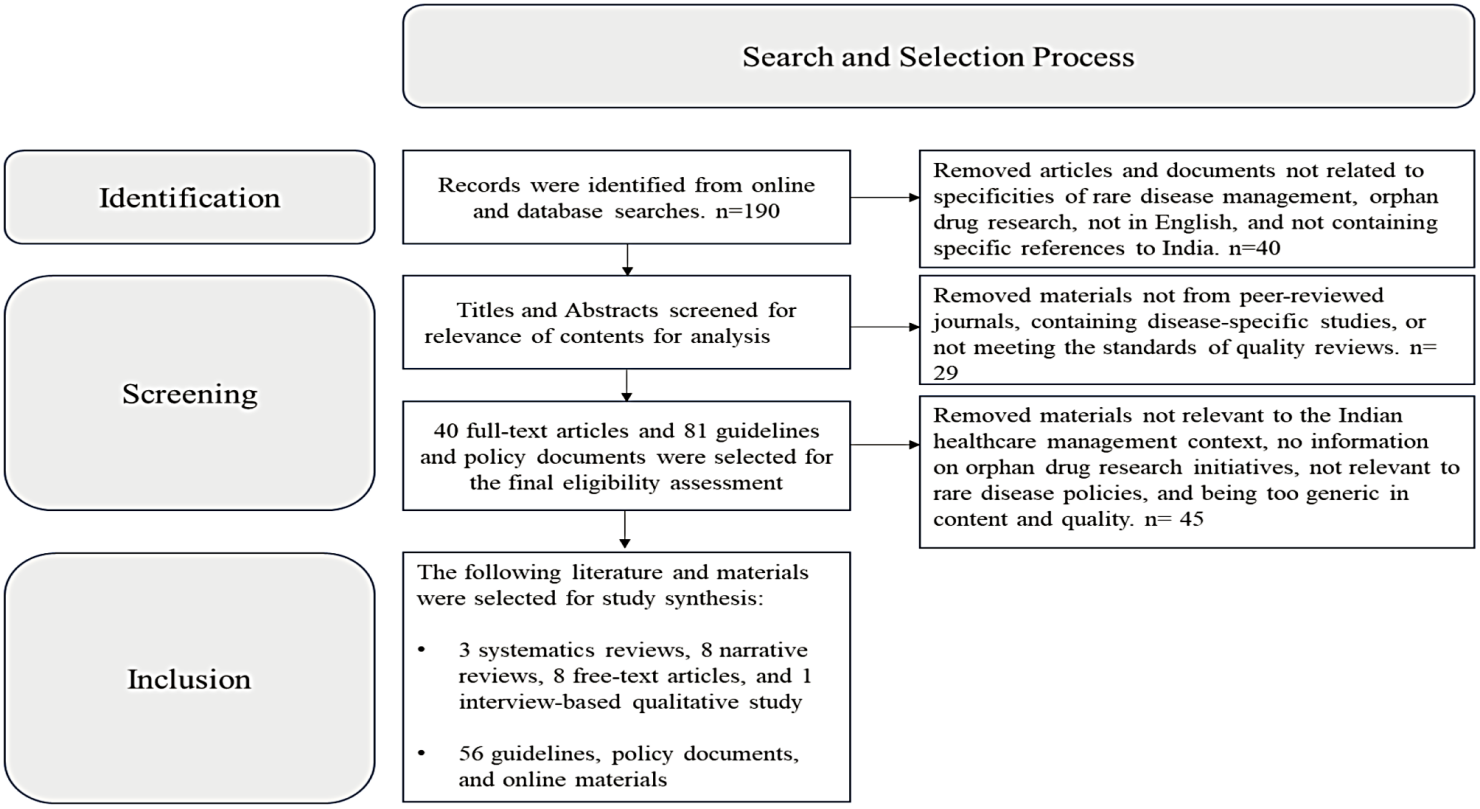
PRISMA flowchart of the search and selection process

Refer to Fig. 1 for an illustration of the search and selection process.

## Results

### Characteristics of the included studies

The key finding from the included studies strengthens the ask for a multi-faceted approach to address India’s rare disease management challenges. There has been significant work undertaken in addressing the rare disease management challenges and assessing the state of orphan drug research and development. A detailed review of the existing literature provided key observations on the nuances of orphan drug development and the rare disease management landscape in India as below:

#### Rare disease management and R&D of orphan drugs

There are existing studies on policy initiatives, the current state of orphan drug clinical trials in India, the lack of participation of Indian patients in global clinical trials, the current state of research and development of orphan drugs undertaken by Orphan Medicinal Product Organizations (OMPOs) and challenges encountered by them, the orphan drug research ecosystem for developing treatments for rare genetic disorders focusing on the genetic and phenotypic diversity of the Indian population, the state of availability of repurposed orphan drugs in India, the role of patient organizations in the rare disease ecosystem of India, the level of awareness about rare diseases among the healthcare providers and researchers in India, and about leveraging the existing National Health Mission infrastructures for implementing a robust rare disease management system [4, 7, 9–14]. These studies identified the key challenge areas within Indian healthcare which encompasses diverse populations, unmapped disease epidemiology, underdeveloped healthcare infrastructure, and lack of skilled resources as the primary problem areas. These studies explored the possibility of augmenting the NHM infrastructure and utilizing the key resources and best practices from these programs to improve rare disease management in India. Studies were also undertaken on the R&D landscape for orphan drug development, the status of repurposed orphan drugs in India, and the key stakeholders in the R&D process. R&D challenges were also discussed and cross-stakeholder collaboration along with adequate funding mechanisms were emphasized. The lack of participation of the Indian population in RD trials was discussed, and issues were identified behind this which include a lack of prevalence data and the absence of financial incentives for marketing orphan drugs in India. Studies were also conducted on the level of awareness among healthcare providers about rare diseases and findings were discussed to identify areas of improvement and techniques to improve the knowledge and awareness of these diseases to ensure correct diagnosis and timely intervention for disease prevention or symptom mitigations.

#### Improving clinical and rare disease registries

One study highlighted the necessity of enhancing the clinical trials registry in India to improve the quality and data reporting timeline to track the conduct of trials on the Indian population. This will help in the timely reporting of ongoing trials and standardize the reporting and data collection protocols for rare disease trials. This information will be crucial for global companies to make informed decisions on the inclusion of Indian patients in Global Clinical trials [15]. One more study analyzed the scope of developing a national rare disease registry based on observations, success stories, and outcomes of the Rare Diseases Registry and Analytics Platform (RD-RAP) of the Asia-Pacific Economic Cooperation (APEC) [16]. The development of a Rare Disease registry based on global standards will help in the timely collection of standardized and high-quality clinical data to support the development of orphan drugs, design and conduct of clinical trials, and regulatory decision-making.

#### Rare disease mission for improving treatment landscape

Two studies focused on implementing a comprehensive rare disease care model and a dedicated nationwide mission initiative, to timely and effectively address the diagnosis and treatment challenges for rare genetic diseases [17, 18]. The papers focused on the importance of PRaGeD (Program on Pediatric Rare Genetic Disorders) and the Comprehensive RD Care Model (CRDC) respectively. These programs aim to improve the detection and diagnosis of rare genetic disorders at an early age to ensure timely intervention, spread awareness, improve data collection and clinical data management, identify genetic variants of a disease, and provide knowledge and resource support to patients and their families.

#### Utilization of existing healthcare resources and digital technology

One more study provides insights into the effective utilization of community healthcare workers and digital technology to track adherence to treatment regimens and assess clinical outcomes [19]. This study analyses the effectiveness of utilizing community healthcare workers and mobile health technologies for tracking medication adherence of cardiovascular disease patients in Kerala, India. This study emphasizes the importance of these resources in ensuring real-time effectiveness and outcome assessment of health interventions. The findings can be beneficial to track outcomes for rare disease patients as well.

#### Clinical research and global rare disease management

Few other studies focused on the global challenges associated with orphan drug clinical research, legislative and regulatory aspects, frameworks associated with rare disease treatment management, pricing challenges, and economic aspects of orphan therapies on rare disease management and the national healthcare budget [1–3, 6, 20–25]. These studies provided valuable insights into the global aspects of rare diseases, the different regulatory approaches to address rare diseases, initiatives undertaken to enhance orphan drug development, identifying innovative clinical trial approaches, undertaking health economics and outcomes research to perform benefit-risk assessment of existing and novel interventions, explores aspects of orphan drug pricing, and identifies areas of improvements and provides key recommendations to improve the research and treatment landscape.

Overall, these studies explored the global aspects of rare disease management and orphan drug research, challenges within the Indian healthcare and research ecosystem to address rare diseases, utilization of existing healthcare programs in India to augment the rare disease management system, and the initiatives taken by orphan drug product companies to address the issue of limited therapeutic access through innovation and development of novel therapies. The key findings from a few of the reviewed studies are provided in Table 2.

**Table 2:** Key findings from the included studies

Study	Research Focus	Aim	Key findings
Richter et al., [1]	To assess the current state of rare disease management and orphan drug development landscape	To explore challenges and opportunities in rare disease clinical trials	Underscores the variations in rare disease definitions globally and emphasizes the importance of harmonizing regulatory considerations for determining prevalence thresholds based on objective criteria
Chakraborty et al.,[11]	To investigate the participation of Indian rare disease patients in global clinical trials for orphan drugs	Understand the reasons for low Indian participation and highlight the importance of inclusion	• Ethnic diversity and affordability impact the participation of patients in clinical trials. • Only 9 out of 63 orphan drugs had trials in India. • Less than 1% of global clinical trials conducted for orphan drugs recruited Indian-origin participants. • Further epidemiological and clinical research is needed to address this gap
Choudhury and Chaube [9]	To study the effectiveness of rare diseases (RDs) management within the context of India’s National Health Mission (NHM)	To assess the potential and limitations of NHM programs in aiding RD care	• Some NHM disease-prevention initiatives address specific RDs and can be expanded to manage preventable RDs. • NHM can play a role in providing a continuum of care for RDs that require lifelong management. However, specialized RD-related treatments are better served in a more focused system.
Chaube et al., [13]	To explore the utility, expandability, and limitation of the Rashtriya Bal Swasthya Karyakram (RBSK) program in the context of rare diseases	To identify how RBSK, a national child healthcare program, can effectively address rare disease management challenges in India	The study identifies that RBSK has immense potential for RD management due to its comprehensive screening, wide target age group, and efficient resource utilization framework and standards. Recommendations are provided to strengthen the program and inspire other low-resource countries.
Pandey et al., [15]	To address the issues in the conduct of clinical trials	To improve the quality and transparency of clinical trial data in India	The clinical trials landscape can be enhanced through an increased registration of clinical trials, better reporting of results, and an improved accountability system
Choudhury and Saberwal [7]	To understand and illustrate the areas of work and the current operations of orphan medicinal product organizations (OMPOs).	To explore the goals, challenges, achievements, and recommendations of OMPOs.	• Most OMPOs are actively involved in the research and development (R&D) of orphan drugs. • Drug development challenges include a lack of funding, supportive government policies, and a conducive research ecosystem. • With suitable policies, OMPOs could scale up and provide relevant products and services to rare disease patients in India.

### Need for the study

Current studies address the challenges faced by the Indian healthcare system in managing rare diseases, the lack of sufficient and quality therapeutic data, and the lack of adequate research on orphan drugs. However, these studies although comprehensive focus more on addressing immediate challenges. It is important to look into the critical aspects of the Indian rare disease scenario more systematically, and holistically and address these issues through an integrated treatment and research ecosystem, to ensure that the solutions are sustainable and flexible. It is not only important to improve healthcare management, but it is also necessary to promote indigenous orphan drug clinical research to increase the affordability of advanced therapies from the context of the Indian rare disease population. This particular study addresses the same by highlighting the current hurdles and proposing solutions by adopting an integrated approach keeping in mind the specificities of the Indian population, policy and healthcare management approaches and priorities, and government initiatives undertaken to improve the rare disease management and orphan drug ecosystem in India.

### Key outcomes of the study

A baseline synthesis of key findings from reviewed studies and guidelines provides a core observation that the rare disease management and orphan drug research landscape needs to be developed around four key areas:

- Understanding disease characteristics and timely identification of anomalies.
- Addressing patient needs and challenges.
- Increasing the availability and affordability of therapies.
- Ensuring a continuum of care through outcome assessments and social and health indicators.

Key insights from existing studies and our assessment imply that although global research and understanding of disease mechanisms through natural history studies provide valuable insights into managing rare diseases and developing orphan drugs, India needs to have a distinct strategy due to its unique circumstances and large ethnic diversity. The policies and guidelines underscore the importance of structured utilization of existing Indian healthcare programs and leverage the expertise and infrastructure to create a dedicated rare disease management system ensuring comprehensive access of patients to quality medical care. The program highlights cover the key aspects of the health missions and programs which provide important information and insights that if leveraged properly can address the rare disease management challenges in India to a considerable extent. In-depth research on prevalent diseases, culturally sensitive trial designs addressing diverse populations within a resource-limited set-up, increased involvement of patients and advocacy groups in policy-making, and collaboration among stakeholders are crucial. Streamlining regulations, dedicated funding, and specialized infrastructure are essential to create a conducive research environment. This approach warrants refinement of scientific research approaches, effective diagnostics, advanced healthcare delivery, adequate budget allocations, and regulatory and financial incentives for orphan drug development specifically targeting the Indian population.

## Discussion

### RD and orphan drug development and accessibility challenges in India

#### Incomplete epidemiological information and disease definition

Insufficient epidemiological data hinders RD prevalence estimates in the Indian population [26]. Extrapolating international estimates of the Indian population is prone to error due to demographic diversity [24]. It is estimated that ~ 96–100 million RD patients are present in India. New Drugs and Clinical Trial Rules (NDCTR) in 2019 released by the Central Drugs Standard Control Organization (CDSCO) defined orphan drugs as therapies to treat diseases affecting less than 500,000 Indian patients [27]. However, the threshold tends to have bias resulting in misrepresentation of the ultra-rare disease population. The threshold may be skewed more towards widely diagnosed and hence prevalent diseases. Thus, a more objective approach is required to arrive at a more accurate threshold encompassing a wide array of therapeutic areas across a wider patient population.

#### Inefficient diagnostic infrastructure

Due to India’s vast population and limited healthcare resources, early detection of RDs is a challenging task. Primary Healthcare Personnel (PHP) often lack adequate knowledge and proper disease awareness, and screening and diagnostic facilities often lack the required infrastructure and skilled resources to conduct comprehensive newborn screening for early detection of disorders. Screening protocols are not standardized and not at par with global standards, which makes even detection at a later stage for progressive disorders a difficult task. On average, RDs in India are diagnosed over a 7-year timeline [28], leading to delayed diagnoses of life-threatening conditions resulting in loss of benefit from available treatments [22]. 

#### Lack of advanced research and development ecosystem

Orphan drug research heavily relies on understanding RD’s natural histories and accessing diverse patient pools to identify disease variants and phenotypic differences across ethnic populations. Research institutions across India have undertaken significant research to identify disease mechanisms, perform genetic mapping, and explore regenerative and advanced therapeutics for treating prevalent rare diseases in the Indian population. However, obtaining long-term treatment and interventional data and analyzing comprehensive clinical information to prepare targeted research plans and strategies is crucial. These face significant hurdles due to infrastructural constraints and sufficient funding, a lack of regulatory incentives, and the absence of dedicated research networks catering to specific therapeutic areas. In India, it’s crucial to implement a good research ecosystem, tailor drug development guidelines to address regional nuances and support safe and effective RD medication and diagnostics research through policy implementation [22]. 

#### Treatment-related challenges related to RDs

- Treatment unavailability.

Currently, many RDs lack effective treatments, with available options often focusing on symptom relief. The scarcity of patient data is a significant challenge, along with limited clinical and diagnostic expertise across healthcare facilities [22, 26]. Healthcare facilities often lack the necessary infrastructure for RD care, especially in remote areas. This, along with a scarcity of skilled resources, makes RD management difficult [23].

b. High cost of treatment.

In India, annual treatment of Lysosomal Storage Disorders (LSDs) using Enzyme Replacement Therapies (ERTs) for a child weighing 10 kg might range from Rs. 2 million ($25,000) to Rs. 25 million ($305,000). A single dose of Zolgensma, a gene therapy product for spinal muscular atrophy, costs Rs. 180 million ($2.1 million) [11, 21]. The high costs of these imported drugs hinder affordable treatment. Lack of insurance coverage and governmental incentives to create a favorable and equitable payer management system cause families to carry the burden of the high cost of treatment. As a result, despite understanding the disease severity and knowing the benefit of an existing treatment, patients and families either withdraw from ongoing treatment or never initiate it, thus restricting patient access to potential benefits and chances of improvement in health condition [20].

c. Competing health priorities in the Indian setting.

RDs impose a substantial economic and resource burden on India since the government has to ensure multiple social sector allocations, thus straining the budget for a comprehensive healthcare system. Prioritizing healthcare resources is challenging since adequate planning to address the varying needs of different patient classes faces significant manpower and budgetary allocation constraints [20]. Competing health priorities and different state policies that vary widely from the aims and objectives of the Government of India’s healthcare initiatives, create policy and legislative hurdles in creating a unified national RD management strategy. Thus, legislation built on policies ensuring equitable access to healthcare for RD patients will create a more sustainable treatment and drug development ecosystem in India [29]. 

### Health policies and programs

RD policy in India is at an early stage of adoption, needing significant refinements and well-laid legislation to enforce and monitor the outcome of recommendations or actions undertaken. Although several initiatives have been taken to address the policy recommendations, outcome studies are lacking to confirm the effectiveness of these initiatives. Additionally, there are Governmental Health Programs and missions to address maternal, child, and adolescent health and specific critical diseases. Some of these programs already address RD aspects related to screening and diagnostics for genetic disorders and birth-related defects. All these programs have varied outcomes across the country based on state-wise infrastructure and resource availability. There are areas of common activities of these programs but each of these serves a different purpose. Outcome assessments of these programs are being conducted but the approach is highly fragmented. The quality and effectiveness of these programs are beyond the scope of this study. An overview of the RD policy and health programs is provided in subsequent sections.

#### National policy for treatment of rare diseases (NPRD)

The Government of India published the NPRD based on expert committee and patient group recommendations in 2021. This policy addresses prevention, awareness, research, diagnosis, treatment, affordable orphan drugs, insurance coverage, and progressive actions [30]. The establishment of 5 National Inherited Diseases Administration Kendras (NIDAN) in India under the Unique Methods of Management and Treatment of Inherited Disorders (UMMID) project aims to strengthen India’s RD diagnostic infrastructure [31]. These centers offer specialized genetic screening. 11 Government Health Facilities have been identified as Centers of Excellence (CoE) for RD diagnosis and treatment, backed by a budget of Rs. 928 million ($11.3 million) [32–34]. Suggested measures include inter-ministerial policy-making, centralized fund allocation, setting up of disease registry, and central and state coordination. The policy also provides an indicative list of diseases prevalent in India and has classified those according to required intervention. Refer to Supplemental File 1 for details.

#### RD registry and research consortium

Refer to Table 3.

**Table 3:** RD registry and research consortium

Organization	Activities
National Consortium for Research and Development on Therapeutics for Rare Diseases (NCRDTRD) [35]	Diagnostic marker development and orphan drug research through collaboration and funding
National Registry for Rare and other Inherited disorders (NRROID) [32, 36]	Collects demography, phenotype, natural progression, and treatment outcomes of RDs

#### Government healthcare programs and missions

Refer to Supplemental File 2 for details. See Table 4 for an overview of the programs.

**Table 4:** Government healthcare programs and missions

Name	Services	Coverage	Activity about RD
Ayushman Bharat - Pradhan Mantri Jan Arogya Yojana (AB-PMJAY)	Annual medical coverage of Rs. 500,000 ($6100) for treatment [37, 38]	Economically disadvantaged patients	No
Rashtriya Arogya Nidhi (RAN)	Financial aid [39] of up to Rs. 5 million ($61,000) at 14 super-specialties [40] Government facilities for RD treatment[41]	Economically disadvantaged patients (not covered under AB-PMJAY) [42]	Yes
Ayushman Bharat Digital Mission (ABDM)	Digital Health ID (ABHA ID), [43] Health Facilities Registry, Telemedicine, Personal Health Records [44, 45]	All Indian citizens, healthcare facilities [46]	No
National Sickle Cell Anemia Elimination Mission	Screening and diagnosis, treatment and management, genetic counseling, research	SCD patients up to 40 years	Yes

#### National health mission programs

The National Health Mission (NHM) [47] under MoHFW and its component programs, aims to achieve universally accessible, accountable, and responsive healthcare to ensure equity, affordability, and quality in Indian healthcare delivery. Refer to Supplemental File 3 for details. Below are the key components of NHM:

#### Reproductive maternal-neonatal-child and adolescent health (RMNCH + A)

Launched in Feb 2013, the RMNCH + A [48, 49] component aims to offer comprehensive, lifelong healthcare, including reproductive health, maternal, newborn, child, and adolescent care, incorporating new interventions and strategies [49]. 

#### Maternal health programs

The MoHFW’s Maternal Health Division runs programs to enhance maternal and newborn care and reduce mortality rates. This strengthens routine health systems through healthcare providers and earmarked treatment facilities [9, 50]. Refer to Table 5.

**Table 5:** MoHFW maternal health programs

Name	Services	Coverage	Activity about RD
Janani Suraksha Yojana (JSY)	Pregnancy detection, Antenatal care, Counseling, and Institutional delivery [50, 51]	Economically Disadvantaged pregnant women [52]	No
Janani Shishu Suraksha Karyakram (JSSK)	Delivery, diagnosis, medications, supplements, pregnancy care, infection management [53]	Pregnant women, Newborns, and infants (up to 1 year) [54]	Yes
Pradhan Mantri Surakshit Matritva Abhiyan (PMSMA)	Antenatal care, congenital defects screening, early-stage diagnosis, birth planning, investigations, medications [55]	Women in their 2nd /3rd trimesters [56]	Yes
Surakshit Matritva Aashwasan (SUMAN)	Newborn Checkups, family planning, congenital anomaly detection, referral services, infection management, and antenatal check-ups [57, 58]	Pregnant women, newborns, and mothers up to 6 months of delivery [59]	Yes

#### Child and adolescent health programs

The NHM child health program aims to improve child survival by integrating interventions addressing infant and under-five mortality [60]. These programs focus on four key areas: Neonatal and Child Health [61, 62], Nutrition [63], Managing Childhood Illnesses, and Immunization. NHM’s adolescent health programs focus on the health, behavior, lifestyle, and psychological development of adolescents. MoHFW has two programs for holistic adolescent development. Refer to Table 6.

**Table 6:** MoHFW child and adolescent health programs

Name	Services	Coverage	Activity about RD
Facility-based Based Newborn Care (FBNC)	Treatment, drugs, diagnostics, and referrals, [9] at Newborn Care Corners (NBCC), Newborn Stabilization Units (NBSU), and Special Newborn Care Units (SNCU) [64]	Sick infants till 15 months after birth	Yes
Rashtriya Bal Swasthya Karyakram (RBSK)	Child Health Screening, [64] tertiary healthcare, and follow-up at District Early Intervention Centers (DEICs) [65, 66]	Children aged 0–18 years [67]	Yes
Integrated Management of Neonatal & Childhood Illnesses (IMNCI)	Home-based care, community outreach, and critical healthcare at First Referral Units (FRUs) [68]	Children less than 5 years old [69]	No
Home-Based New-Born Care (HBNC)	Home-based care, birth planning, vaccinations, referral, and counseling [61]	Sick infants till one year after birth [70]	Yes
Home Based Young Child (HBYC)	Breastfeeding, immunization, weight management, and nutrition counseling [9, 62]	Children of 3–15 months of age [71]	Yes
Rashtriya Kishor Swasthya Karyakram (RKSK)	Nutrition, sexual and reproductive health, mental health, counseling, diagnosis, and treatment [72]	Adolescents in the age group of 10–19 years [73]	Yes
School Health & Wellness Programme (SHWP)	Health Screening, Electronic Health Records (EHRs) [74]	Children aged 8–18 years [75]	No
New Born Screening (NBS)	Neonatal screening for deafness, blindness, genetic disorders, and defects at birth	All newborns/high-risk births/neonates with a family history of disorders	Yes

#### Information systems for data collection

Refer to Supplemental File 4 for details. Refer to Table 7.

**Table 7:** Data collection systems

Name	Provision
Maternal Death Surveillance and Response (MDSR)	Identification, notification, review, and prevention of maternal deaths. [76]
Reproductive and Child Health (RCH) Portal/Mother and Child Tracking System (MCTS) Portal	Reproductive health services and benefits tracking for women and children up to 5 years. [76, 77]
Mother And Child Protection Card (MCP)	Pregnancy care tracking, immunization, breastfeeding, and growth tracking. [76, [78]

### Integrated RD management and orphan drug development approach

**Fig. 2:**
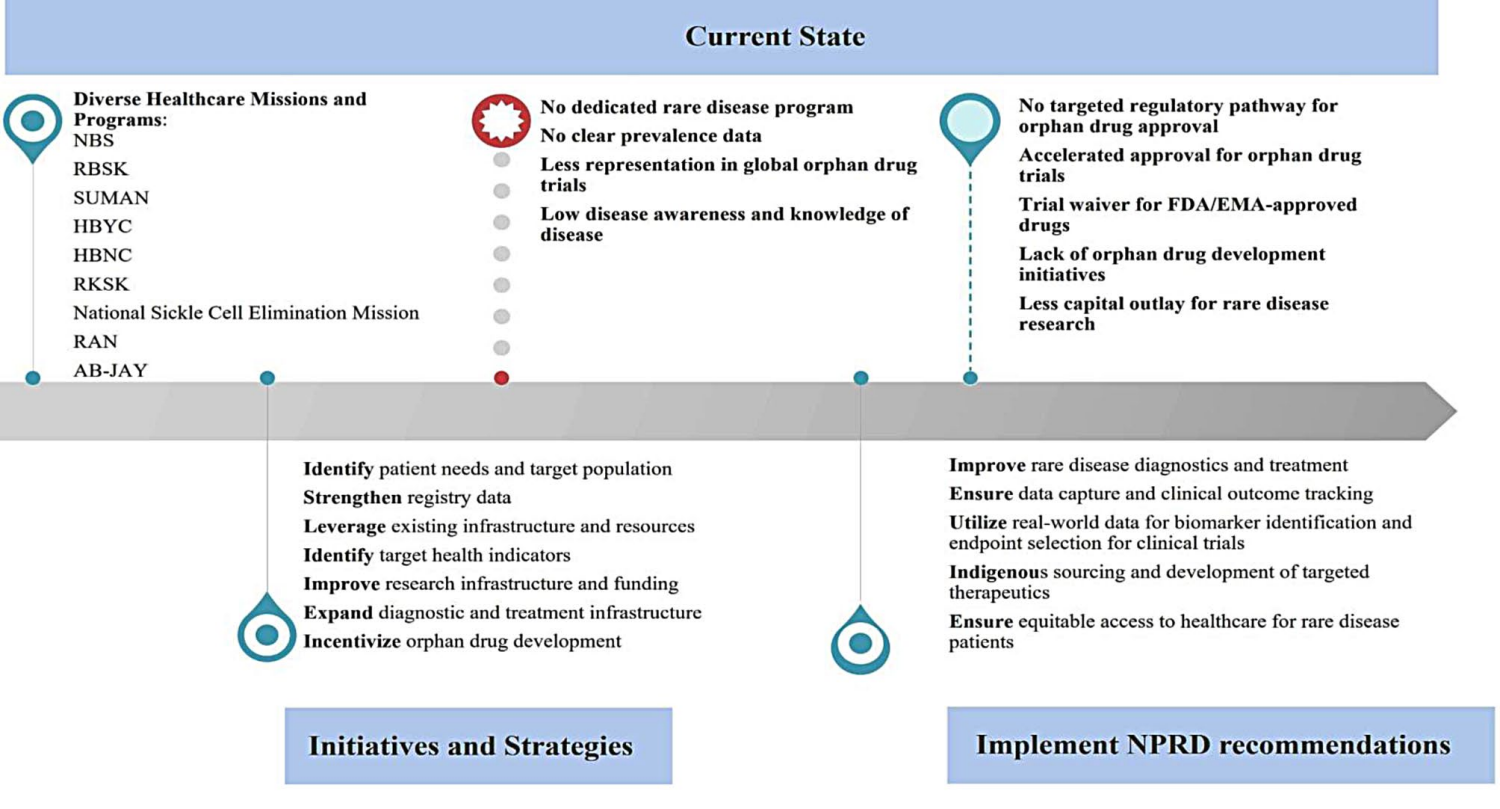
State of health programs, rare disease treatment management, and orphan drug development in India

**Fig. 3:**
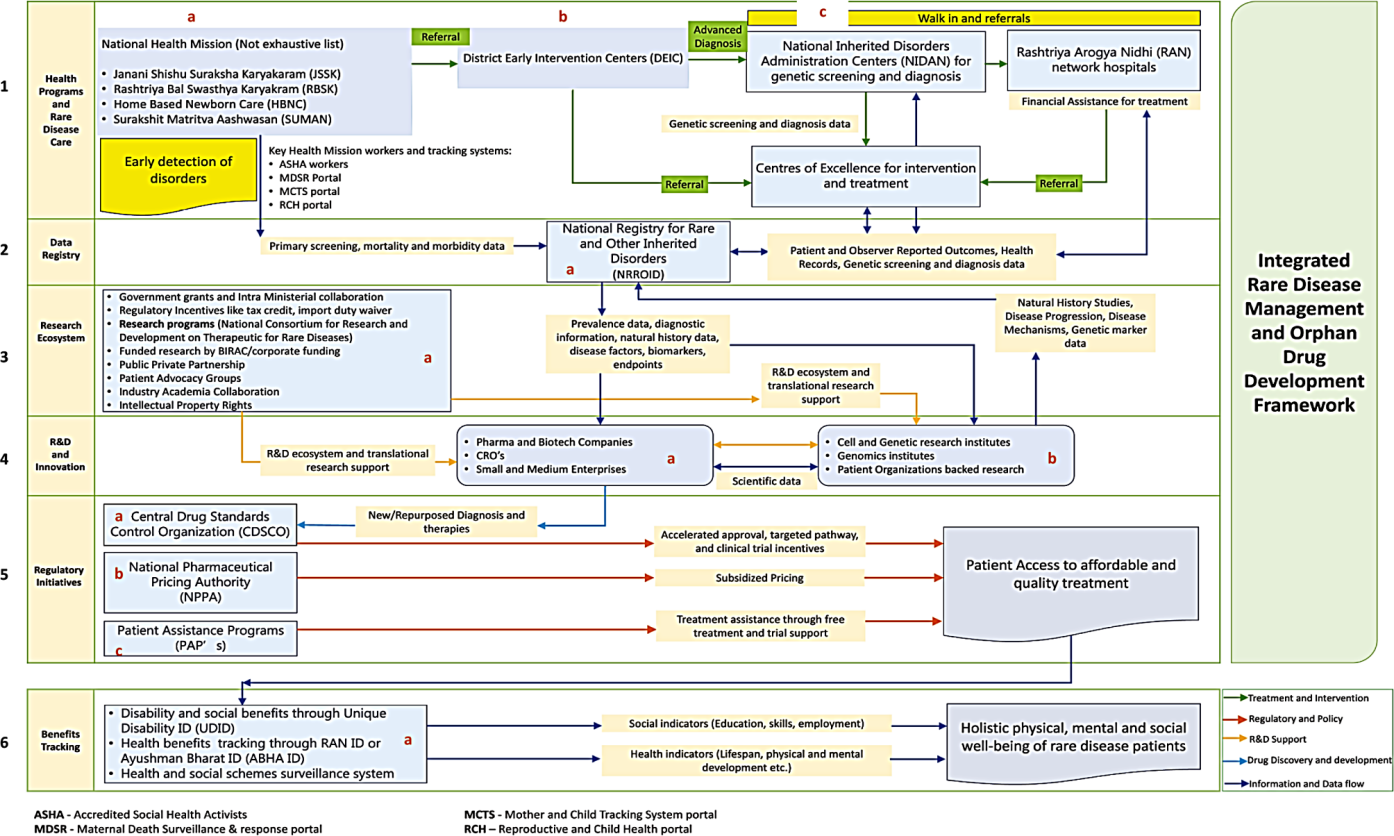
Integrated RD management and orphan drug development framework

In addressing RDs in India, establishing effective diagnostic and treatment strategies is crucial. This requires a robust infrastructure, specialized centers, well-trained healthcare professionals, and comprehensive data management. Affordable therapies, with transparent insurance and government support, will ensure lifelong patient care and inclusivity. Indigenous drug development can improve availability. NPRD emphasizes genetic screening, mental and physical well-being, maternal counseling, and ongoing intervention. Leveraging existing healthcare programs is a practical approach to building an integrated RD treatment ecosystem in India, considering resource constraints. The state of health programs and the ideal RD management plan are depicted in Fig. 2. The recommended approach for integrated RD management and orphan drug research is shown in Fig. 3.

### Health programs and rare disease care

#### Primary and secondary detection of disorders and prevention

The NHM programs (JSSK, RBSK, SUMAN, etc.) have made significant progress, and consist of robust infrastructure and trained workers. Few of these constituent programs have components of early detection of rare disorders and diagnosis. Refer to Table 4, Table 5, and Table 6. The healthcare resources from these programs can be used for genetic counseling, promoting safe pregnancy, and early detection of genetic disorders [9]. Any anomalies detected during childbirth or routine care can be referred to DEICs for further screening and intervention. This approach may increase survival chances for patients suffering from life-threatening conditions like Duchenne Muscular Dystrophy (DMD), due to timely detection and initiation of treatment. Accredited social health activists (ASHA) workers and auxiliary nurse midwives (ANM) can play crucial roles based on their experiences administering key action areas of these programs. These will address the key requirements of NPRD. Refer to Part 1(a) of Fig. 3. It is to be kept in mind that ASHA workers are compensated using an incentive method. Given the geographic spread of India, healthcare infrastructure in many parts of the country is underdeveloped. In these areas, the ASHA workforce needs to be expanded with proper training to identify birth-related disorders and to conduct periodic sessions to spread awareness of rare diseases. Early detection and prevention are the best options for preventing genetic diseases and through proper practical skills training programs and leveraging the NHM infrastructure, rare disease management can be strengthened to a significant extent. Primary screening, mortality, and morbidity data collected from these sources are fed into MCTS, MCTS, and RCH portals. This information can be further analyzed and refined through advanced analytical techniques and customized for the NRROID.

#### Diagnosis, genetic screening, and access to RD treatment

Referrals from DEICs will be handled by NIDAN kendras, CoEs, and RAN hospitals, employing advanced genetic screening for the diagnosis of rare disorders. Treatment regimens in the specialty centers should be tailored based on disease progression, severity, and indications utilizing the IMNCI methodology. This method uses an evidence-based case study approach and allows for customized treatment planning to address the specific needs of patients based on manifestations and diagnostic outcomes. Staff training, coordination, and knowledge sharing enhance understanding of RDs, which helps identify patient responses to treatments. Another approach that can be adopted is collecting drug utilization and treatment data from clinical pharmacists. In India, pharmacists play a crucial role in the healthcare system. They can help track drug usage, and outcome progression, and refer anomalies to clinicians on behalf of patients. To implement this, enhancing the role of pharmacists is required through proper legislation, comprehensive training should be provided, and specialized skills and qualifications should be made mandatory for specific roles. Implementing the Comprehensive RD Care Model (CRDC) for screening and diagnosis at specialized healthcare facilities is crucial. The CRDC was started at the Centre for Human Genomics and Counselling (CHGC) at JSS Medical College and Hospital, Mysuru, India. Its success stories, operating model, and best practices can serve as a benchmark for other facilities [17]. Genetic screening, diagnostic, and clinical outcome data are valuable information for the NRROID and other disease registries. This information collected from the advanced healthcare facilities will be crucial to support drug development and trial designs. Refer to Part 1(b) and 1(c) of Fig. 3.

### Data registry

The ICMR established the National Registry for RDs to compile patient experiences and treatment outcomes. Data, collected digitally from various healthcare facilities, including primary centers and hospitals, early-stage diagnosis, and morbidity and mortality data from MCTS, RCH, and MDSR databases can help create demographic maps and compile epidemiological information. The NRROID currently has 13,972 records covering 12 disease areas. Please refer to Table 8 below:

**Table 8:** NRROID disease areas and available records [36]

Disease Area	Number of Records
Fabry Disease	37
Thalassemia	3466
Glycogen Storage Disorder	78
Bleeding Disorder	1038
Inborn errors of metabolism (IEM)	840
Pompe Disease	79
Sphingo Lipidosis	754
Skeletal Dysplasia	367
Neuromuscular Disorders (NMD)	5794
Mucopolysaccharidosis	555
Small Molecule	264
Primary immune deficiency diseases (PID)	700

The registry development faces multiple challenges including a lack of standardized data collection and reporting methodologies, underdeveloped technical infrastructure, poor awareness among stakeholders, data quality standards are not up to date, lack of funding, and no visibility of target usage of the information for R&D purposes [16]. The registry is also not well-organized and cataloged to make it user-friendly. The disease areas reported are much lower than what has been estimated to be prevalent in India. The current information is insufficient to provide a proper epidemiological landscape and perform statistical analysis of clinical data. It is important to ensure that clinical data is collected in real-time, adequate data quality checks are undertaken, and relevant information is extracted to support R&D, undertake health economic assessments, analyze treatment outcomes, and develop a proper payer management system to cover treatment expenses for rare diseases. In regulated countries like the USA and the EU, centralized and disease-specific registries are maintained utilizing stringent data governance frameworks. In India, similar approaches are required through adequate administrative oversight, technical expertise, usage guidelines, access management, and privacy regulations. These supported by standardized data collection methods using trained healthcare providers can create a valuable pool of real-world data, biomarkers, and treatment endpoints to support rare disease trial designs and orphan drug development. This data will also help in healthcare planning linked to a common health identifier like ABHA ID [44, 45], and integrated through Unified Health Interface (UHI) [79] can empower a digital health system, attract investment, and ensure a seamless continuum of care [54]. Refer to Part 2(a) of Fig. 3.

### Research ecosystem

#### Research initiatives

India’s orphan drug development can be supported by open innovation, Biotechnology Industry Research Assistance Council (BIRAC) funding, academic and corporate partnerships, CoEs for drug development, and clear policies to ensure consistent investment. Inter-sectoral collaboration, manufacturing incentives, tax relief on R&D expenses, R&D grants, data exclusivity, and Intellectual Property protection can boost research and indigenous orphan drug development. Major companies like AstraZeneca and Novartis have initiated RD trials in India due to improved regulatory conditions [80]. A robust clinical trial ecosystem supported by adequate funding will expedite custom therapeutics, lowering costs and improving access to innovative therapies. Research on biosimilars and repurposing existing drugs to treat orphan indications will ensure timely and low-cost access to critical medicines for Indian patients [81]. Another important player in this ecosystem is patient advocacy groups. The patient advocacy groups can help identify target patients for clinical trials and treatment assistance, support research initiatives, and help frame and refine rare disease policies [12]. A few of the initiatives taken by GoI are provided in Supplemental File 5. Refer to Part 3(a) of Fig. 3.

#### R&D and innovation

The ICMR established the NCRDTRD to promote translational R&D of innovative therapies for rare diseases. NCRDTRD can bring together cell and genetic research institutes, academic research institutions, regulatory and policy research centers, and patient organizations to work collaboratively on natural history studies, disease progression mapping, genetic mapping, biomarkers, and endpoint generation for rare diseases. All the scientific data generated can be shared with clinical research organizations (CROs), pharmaceutical and biotechnology companies, and startups, either directly or through centralized data-sharing mechanisms from NRROID. These organizations will also receive valuable prevalence, diagnostic, and demographic information from NRROID to undertake targeted therapeutic development and clinical trial designs. The data from these organizations and NCRDTRD partners can further be fed back into NRROID or specific-purpose scientific databases to undertake further research and support regulatory decision-making. Refer to Parts 4(a) and 4(b) of Fig. 3.

### Regulatory initiatives

#### Regulatory pathway

A dedicated regulatory pathway should be established to bolster orphan drug development and expedite approval, by adopting best practices from the USA or the EU. This pathway can grant exclusive rights and tax incentives, encouraging more players to develop orphan medicines in India. The launch of rare disease programs under the ambit of CDSCO and the MoHFW will further strengthen the mission of targeting prevalent diseases and foster innovative regulatory approaches to assess marketing authorization applications for orphan drugs. Utilizing conditional approval systems can help introduce breakthrough therapies for critical patients and based on outcomes further regulatory assessments can be undertaken for final decision-making. Adopting novel technologies like AI for regulatory assessments can reduce the timelines and accelerate the introduction of novel therapies. Refer to Part 5(a) of Fig. 3.

#### Pricing of orphan drugs

The Ministry of Chemicals and Fertilizers (MoCF) amended the Drugs Prices Control Order (DPCO) 2013, exempting newly patented drugs (including orphan drugs) that were developed outside India from pricing regulations for five years [13]. This is enforced by the National Pharmaceutical Pricing Authority (NPPA). This amendment aimed to encourage and incentivize domestic orphan drug development and encourage global companies to market approved products in India [82]. The New Drug Exemption order, although an important step to improve the research landscape in India may indirectly lead to high prices of critical drugs. It has also been observed that many companies have attempted to launch products without applying for exemption and as a result, domestic manufacturers are facing stiff competition to develop therapies [83]. A multi-dimensional approach for determining orphan drug prices is needed using pharmaco-economical studies, clinical outcome-based assessments for value-based pricing, volume-based pricing based on government purchase agreements, variable pricing based on target orphan indication, and extending custom duty waivers for life-saving therapies [20, 84]. Refer to Part 5(b) of Fig. 3.

#### Patient assistance programs (PAPs)

PAPs play a crucial role in supporting initial access to high-cost therapies. A few of the key programs are the Managed Access Program (MAP) for certain unapproved or investigational treatments by Novartis, the Blue Tree Program by Roche India, Pfizer PAP India by Pfizer, and Sparsh by Dr. Reddy’s Laboratories. Augmenting the PAPs can improve access to conditionally approved therapies and help to cover more disease areas. PAPs can also be initiated by patient organizations, in line with the National Organization for Rare Disorders (NORD) PAPs in the USA. Crowdfunding and public-private donations can help to fund these programs. Enhancing PAPs is crucial since the outcomes from these programs can provide valuable data and infrastructure for effectiveness assessments and undertaken observational and pragmatic clinical trials. Refer to Part 5(c) of Fig. 3.

### Benefits and outcome tracking

#### Rehabilitation initiatives, inclusive societal system, and well-being

To address genetic and birth-related anomalies, the government introduced disability benefits through the Rights of Persons with Disabilities (RPWD) Act, 2016. The Unique Disability ID (UDID) via Swavlamban card aids in tracking and ensuring access to social benefits [85]. The government can foster inclusive education and strengthen healthcare for children with special needs, tracking outcomes via social indicators like skills, education, and employment, in alignment with NPRD mandates for holistic healthcare delivery and inclusivity [9]. Refer to Part 6 (a) of Fig. 3.

#### Implementation of a centralized surveillance system

For effective RD management, precise genetic screening and data collection are essential to identify prevalent patterns in various communities and develop targeted treatment regimens. The diagnostic and clinical outcomes need to be tracked and maintained to assess the state of services and quality of life. A Centralized surveillance system, linked to the National Registry via ABHA ID, can facilitate seamless treatment management and continuous monitoring of a patient’s quality of life, lifespan, and health. This data will provide essential epidemiological information to orphan drug research organizations and pharmaceutical companies to design drug development blueprints and clinical trials. Refer to Part 6 (a) of 3.

### Recommendations

#### Financial assistance

Financial assistance for RD treatment provided at RAN network hospitals and CoEs can be increased as the budget al.location and coverage provided as assistance is insufficient to address the diagnostic and treatment expenses. Insurance companies need policy support and transparent regulations to cover RD treatments, as insurance coverage does not apply to many life-threatening diseases. Combining government funds with insurance coverage can alleviate financial constraints for patients to a greater extent. Dedicated financial sources and a proper payer management framework are crucial for administering quality care. Patient organizations have an important role in sourcing treatment assistance for needy patients. Similar organizations in other geographies such as the USA and the EU actively coordinate with payers to obtain necessary approvals, run PAPs, and arrange crowd-funding to support treatment costs [12]. 

It is also important to enhance the budgets of NHM programs to train and deploy adequate resources and augment the infrastructure of Maternal and Newborn Screening (NBS). In FY 2024-25, the GoI has allocated Rs. 36,983 crore or USD 451 million for the NHM programs [86]. Given the importance of these programs in detecting inborn disorders and reducing maternal and child deaths, this budget seems inadequate looking at the span of these programs. NBS is still underdeveloped in India with several identified disorders still not covered or are highly expensive making it difficult for healthcare facilities to build the required infrastructure or to train the required resources for these screenings. It is important that either the Government augment the budgets or earmark separate funds for diagnostic and genetic screening to facilitate early detection of disorders and treatments of the same.

#### Leveraging real-world evidence for clinical trials, drug development, and regulatory approvals

Existing maternity and newborn data collection systems and treatment and diagnostic data from RAN network hospitals, DEICs, NIDAN kendras, and CoEs provide essential clinical data, including Patient Reported Outcome (PRO) and Clinician Reported Outcome (CRO), pharmaco-epidemiological, and demographic data, enabling the generation of Real-World Evidence (RWE). RWEs will aid in identifying biomarkers, developing clinically relevant endpoints, supporting targeted treatment development, and improving diagnostic capabilities. RWE from clinical outcome assessments can identify pharmacological patterns across different physiology. This will help develop custom or personalized therapeutics based on disease variation and physiology. This capability is also essential to designing innovative clinical trials for the Indian rare disease population. Due to the demographic, ethnic, and genotypic diversity of patients, innovative trial designs like adaptive trials, basket trials, umbrella trials, and pragmatic and observational trials can be beneficial in the Indian context and increase their inclusivity in global clinical trials. Regulatory decisions can also leverage RWE and clinical outcome data from innovative trials to accelerate the approval timelines.

#### RD awareness

Government-led initiatives for RD awareness are essential, with ASHA workers and ground-level staff pivotal in providing critical disease and diagnostic information to patients and their families, facilitating healthcare access from an early stage to prevent progression, and reporting high-risk cases to proper healthcare providers on time. It is essential to design training on the latest developments in rare disease diagnostics, management, and outcome tracking for healthcare professionals in collaboration with the National Institute of Health and Family Welfare (NIHFW). Organizing public awareness events, engaging NGOs in designing disease-specific awareness programs, and empowering patient advocacy groups are additional steps that can be undertaken to ensure timely information is available to healthcare providers and patients. This information can be leveraged to design treatment plans that are effective, adaptable, and scalable based on the nature and outcome of the disease [14]. Similar initiatives should be undertaken for clinicians and tertiary healthcare providers to improve effectiveness in treatment planning, delivery, and outcome assessments. They should also be trained in data reporting and Electronic Health Records (EHR) management for digital health tracking and remote health management.

#### Regulatory framework for RD clinical trials, augmenting clinical trial registry and orphan drug approvals

As per NDCTR, innovative drugs approved in the USA, EU, Australia, Canada, and Japan can bypass Phase 2/3 trials, securing direct marketing authorization in India, thus ensuring faster access to life-saving therapies. Accelerated approval of 90 days is being implemented for therapies developed domestically. Despite these initiatives, orphan drug availability in India is not as expected. Although there is a definition of orphan drugs in India, a separate classification still doesn’t exist. A few orphan drugs approved in India based on FDA classification are Cannabidiol for the treatment of Lennox-Gastaut syndrome, Pralsetinib for the treatment of adults with metastatic RET fusion-positive non-small cell lung cancer (NSCLC), and Tepotinibe for the treatment of metastatic non-small cell lung cancer with MET exon 14 skipping mutations. The participation of Indian patients is insufficient to encourage the marketing of innovative therapies to Indian patients. Even if the exemption criteria will enable pharmaceutical manufacturers to launch globally approved orphan drugs in India, safety surveillance and effectiveness studies should be strengthened to ensure the drugs are safe for the Indian population. Genetic and pharmacological studies should be performed concurrently to ensure the drugs are effective for all molecular subsets of the diseases in the Indian population. These data can be used by domestic organizations to further develop tailored therapies.

The Clinical Trial Registry of India (CTRI) should be augmented in line with global trial registries to ensure syncing with international platforms, ease of access, data management, and analysis of trial data [15]. This will help improve trial designs, planning, and timely assessment of clinical data for decision-making. The information in this registry can also be used as external controls or synthetic control arms to support innovative RD trials and observational studies. It is crucial to overcome current challenges to ensure clinical trials are seamlessly conducted for rare diseases, derive meaningful clinical outcome information, and support regulatory decision-making.

#### Public private partnerships (PPPs) in RD research

To bridge the gap in RD research, Public-Private Partnerships (PPPs) are crucial. PPPs bring together academia, NGOs, philanthropists, government agencies, and private sector entities to dedicate funds and allocate skilled resources toward developing innovative therapies for rare diseases. Establishing an orphan drug research PPP fund can provide financial resources to advance preclinical and clinical research on RDs [87]. This can be achieved by ensuring ongoing governmental support and developing targeted orphan drug research and development policy that will facilitate researchers with grants to undertake natural history studies, new therapy development, development of clinically relevant endpoints, and design clinical studies targeting Indian patients [25]. The Ministry of Science and Technology announced 75 AMRIT grants in 2021 under the DBT-BIRAC for Biotech Startups, Industries, Academia, and Research Bodies under the Public-Private Partnership mode to undertake high-quality interdisciplinary research to support innovative drug development. The amount of grant that can be availed will be between Rs 10–15 Crore over two to three years [88]. Further initiatives are required in this area by involving corporate houses as part of their CSR initiatives and launching scientific grant programs through targeted budget al.locations.

## Conclusion

India faces significant economic and healthcare challenges in addressing RDs. Orphan drug development has lagged, due to regulatory gaps, lack of RD patient data, limited incentives for domestic manufacturing, and market uncertainties. Although NPRD aims to tackle these issues, its implementation remains unclear. To overcome these hurdles, leveraging existing healthcare programs and infrastructure utilizing existing resources for genetic counseling, diagnosis, and affordable treatment is essential. Key policy measures include establishing an integrated research ecosystem, incentivizing orphan drug research, creating patient registries, tailored clinical trial policies, patent protection, and streamlined approvals. Standardized protocols, epidemiological studies, regulatory reforms, and improved diagnostics are crucial for effective RD management. Targeted genetic counseling, prenatal screening, and early diagnosis can significantly enhance outcomes. Increased participation from global and Indian pharmaceutical companies and startups through favorable policy initiatives and research incentives could develop a comprehensive RD management and orphan drug development ecosystem, making RD treatments more affordable for Indian patients.

## Data Availability

Data sharing is not applicable since no data analysis was performed and no datasets were generated, as this work was undertaken using a theoretical approach.

## Abbreviations

ABDM: Ayushman bharat digital mission
ABHA: Ayushman bharat digital health ID
AB-PMJAY: Ayushman bharat - pradhan mantri jan arogya yojana
ANM: Auxiliary nurse midwives
ASHA: Accredited social health activists
CDSCO: Central drugs standard control organization
CHGC: Centre for human genomics and counselling, jss medical college, mysuru
CoE: Centers of excellence
CRO: Clinician reported outcome
DEICs: District early intervention centers
DMD: Duchenne muscular dystrophy
EHRs: Electronic health records
ERTs: Enzyme replacement therapies
FBNC: Facility-based newborn care
FRUs: First referral units
GoI: Government of India
HBNC: Home-based new-born care
HBYC: Home based young child
ICMR: Indian council of medical research
IMNCI: Integrated management of neonatal & childhood illnesses
JSSK: Janani shishu suraksha karyakram
JSY: Janani suraksha yojana
LSDs: Lysosomal storage disorders
MCTS: Mother and child tracking system
MDSR: Maternal death surveillance and response
MoHFW: Ministry of health and family welfare
NBCC: Newborn care corners
NBS: New born screening
NBSU: Newborn stabilization units
NCRDTRD: National consortium for research and development on therapeutics for rare diseases
NDCTR: New drugs and clinical trial rules
NIDAN: National inherited diseases administration kendras
NIHFW: National institute of health and family welfare
NPRD: National policy for treatment of rare diseases
NRROID: National registry for rare and other inherited disorders
PHP: Primary healthcare personnel
PMSMA: Pradhan mantri surakshit matritva abhiyan
RAN: Rashtriya arogya nidhi
RBSK: Rashtriya bal swasthya karyakram
RCH: Reproductive and child health
RD: Rare disease
RKSK: Rashtriya kishor swasthya karyakram
RMNCH+A: Reproductive maternal-neonatal-child and adolescent health
RPWD: Rights of persons with disabilities act
RWE: Real-world evidence
SHWP: School health & wellness programme
SNCU: Special newborn care units
SUMAN: Surakshit matritva aashwasan
UDID: Unique disability ID
UHI: Unified health interface
UMMID: Unique methods of management and treatment of inherited disorders
